# *Salmonella* Paratyphi A Rates, Asia

**DOI:** 10.3201/eid1111.050168

**Published:** 2005-11

**Authors:** R. Leon Ochiai, XuanYi Wang, Lorenz von Seidlein, Jin Yang, Zulfiqar A. Bhutta, Sujit K. Bhattacharya, Magdarina Agtini, Jacqueline L. Deen, John Wain, Deok Ryun Kim, Mohammad Ali, Camilo J. Acosta, Luis Jodar, John D. Clemens

**Affiliations:** * International Vaccine Institute, Seoul, Korea; † Guangxi Centers for Disease Control and Prevention, Nanning, People's Republic of China; ‡ Aga Khan University, Karachi, Pakistan; § National Institute of Cholera and Enteric Diseases, Kolkata, India; ¶ National Institute of Health, Research and Development, Jakarta, Indonesia; # Sanger Institute, Cambridge, United Kingdom

**Keywords:** epidemiology, infectious diseases, enteric diseases, Salmonella paratyphi A, dispatch

## Abstract

Little is known about the causes of enteric fever in Asia. Most cases are believed to be caused by *Salmonella enterica* serovar Typhi and the remainder by *S*. Paratyphi A. We compared their incidences by using standardized methods from population-based studies in China, Indonesia, India, and Pakistan.

Enteric fever still causes substantial illness and death in many parts of the world, especially in poorer nations. *Salmonella enterica* serovar Typhi is believed to cause most enteric fever episodes, and a smaller portion are caused by *S*. Paratyphi (*1**–**3*). This assumption, however, may no longer be true. Since 1999, more *S*. Paratyphi A than *S*. Typhi strains have been isolated in the province of Guangxi, southeastern China (*4*). Increasing isolation rates of *S*. Paratyphi A has also been reported from India (*5*). This finding has 2 major implications for the prevention of enteric fever. First, licensed typhoid fever vaccines (Vi polysaccharide and live oral Ty21a) do not protect against infections caused by *S*. Paratyphi A, and they may become less useful in controlling enteric fever in regions of Asia. Second, transmission and risk factors for *S*. Typhi and *S*. Paratyphi are different in Indonesia (*6*), so reduction strategies effective against *S*. Typhi may not protect against *S*. Paratyphi. Since little is known about the current cause of enteric fever in Asia, we compared *S*. Typhi and *S*. Paratyphi A incidence from study sites in China, Indonesia, India, and Pakistan by using standardized epidemiologic and laboratory methods (*7*).

## The Study

After a baseline census, surveillance was conducted in study sites in Karachi, Pakistan; Calcutta, India; North Jakarta, Indonesia; and Hechi City, China, for 12 months to identify typhoid and paratyphoid cases from specific populations at high risk (Table). None of the sites had specific enteric fever control programs in the past. Hechi City, China, is located in Guangxi Zhuang Autonomous Region where Vi vaccines had been used in the past (*8**,**9*); however, no such intervention had taken place in Hechi City. The closest county with a vaccination program was ≈80 km away and vaccinated only students (29,000 doses in 2001).

**Table:** Population and enteric fever episodes in 4 Asian countries

Country	Pakistan	India	Indonesia	China
Site	Karachi	Calcutta	North Jakarta	Hechi City
Surveillance period	Aug 2002–Jul 2003	Sep 2003–Aug 2004	Aug 2002–Jul 2003	Aug 2001–Jul 2002
Age group surveyed (y)	2–16	All ages	All ages	5–60.9
Population under surveillance (no.)	15,219	57,075	160,257	98,376
Total enteric fever cases	71	102	154	42
No. *Salmonella enterica* serovar Typhi cases (%)	60 (85)	78 (76)	132 (86)	15 (36)
No. *S*. Paratyphi A cases (%)	11 (15)	24 (24)	22 (14)	27 (64)

During the surveillance period, persons with fever who lived in each study area were requested to visit participating healthcare providers. We collected 5–10 mL blood from adults with fever >3 days' duration into Bactec bottles (Becton Dickinson, Franklin Lakes, NJ, USA). We collected 3–8 mL from children with fever >3 days' duration into Pediatric Bactec bottles. The bottles were incubated at 37°C for 7–10 days and visually checked for growth every day. Bottles were subcultured on MacConkey agar on days 1, 2, 4, and 7 or when turbidity was detected. Suspected colonies were screened by using Kligler iron agar, sulfide-indole-motility medium, urea agar, and citrate. Colonies that showed biochemical reactions suggestive of salmonellae were confirmed serologically by Felix-Widal tube agglutination test with specific O and H antisera (Becton Dickinson). All *Salmonella* isolates were confirmed at a reference laboratory (University of Oxford, Wellcome Trust Clinical Research Unit, Ho Chi Minh City, Vietnam).

Incidence rates were calculated by using age-specific denominators of the population living in the catchment area based on the study census. We assumed that each person living in the study area contributed 12 months of person-time to the denominator. The number of disease episodes in eligible individuals was used as the numerator.

Each study received individual approval from the local ethical committees, the institutional review board of the International Vaccine Institute (Seoul, Korea), and the Secretariat Committee for Research Involving Human Subjects, World Health Organization (Geneva, Switzerland).

During the surveillance period, 285 *S*. Typhi episodes and 84 *S*. Paratyphi A episodes were detected at the 4 sites (Table). In Indonesia, 14% of enteric fever episodes were caused by *S*. Paratyphi A, in Pakistan 15%, in India 24%, and in China 64% (Figure). The highest *S*. Typhi incidence was observed in Pakistan (394/100,000/year), and the lowest *S*. Typhi incidence was found in China (15.2/100,000/year). The highest *S*. Paratyphi A incidence was also seen in Pakistan (72/100,000/year), and the lowest *S*. Paratyphi A incidence was seen in Indonesia (13.7/100,000/year).

**Figure:**
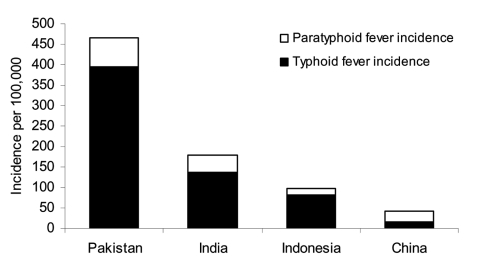
Incidence of Salmonella enterica serovar Typhi and S. Paratyphi A in 4 Asian countries.

## Conclusions

The perception that a small proportion of enteric fever cases are caused by *S*. Paratyphi A is probably no longer true in many regions of Asia, especially in southeast China, where *S*. Paratyphi A is already more frequently isolated than is *S*. Typhi. This finding could be signaling the emergence of *S*. Paratyphi A as a pathogen in Asia. Comparison of *S*. Paratyphi A incidence during the last decade is needed to prove this hypothesis. However, none of the sites have comparable surveillance data on *S*. Paratyphi A over time. An alternative explanation is that the incidence of *S*. Typhi is decreasing. Previous reports from vaccine trials have shown successful control of *S*. Typhi but no changes in the incidence of *S*. Paratyphi A (*8**,**10**,**11*). Nonetheless, the reversal of the proportion of *S*. Typhi and *S*. Paratyphi A infections in Hechi City, China, is unlikely to be the result of typhoid fever control in other counties, considering the distance and oral-fecal transmission route of *S*. Typhi and *S*. Paratyphi A.

Economic growth in Asia has also resulted in improved water supply, sanitation, and hygiene; however, we have no reason to assume selective reduction only for transmission of *S*. Typhi. The incidence of typhoid fever per 100,000 in the different countries follows the same pattern as mean gross national income (2003) and the mortality ranking for children <5 years (*12*).

The sensitivity of blood culture for *S*. Typhi is well described, but little is known about *S*. Paratyphi A. Bacterial loads during infection are probably similar for both *S*. Typhi and *S*. Paratyphi A (J. Wain, unpub. data). Furthermore, *S*. Paratyphi A had been rarely isolated in these regions, which suggests that the increase in isolation rate of *S*. Paratyphi A is possibly caused by an increase in the number of cases of enteric fever caused by *S*. Paratyphi A rather than any bias toward blood culture–positive disease.

Besides the limitation that our studies only describe a 12-month period, the population varied between sites. In Pakistan, only children 2–16 years of age were included. As *S*. Paratyphi A infections are more frequently observed in adults, including older patients in surveillance may increase *S*. Paratyphi A incidence rates reported from Pakistan.

In China and India, countries with the largest populations in the world, *S*. Paratyphi A is the causal agent for a substantial proportion of enteric fever episodes that cannot be distinguished clinically from typhoid fever episodes. While similar treatment strategies may work for both organisms, future enteric fever prevention strategies in Asia must focus on *S*. Paratyphi A as well as on *S*. Typhi, especially when considering the emergence of drug-resistant strains (*13**–**15*). Future vaccination strategies should include bivalent vaccines that protect against *S*. Typhi as well as *S*. Paratyphi A. Otherwise, the protective effectiveness of typhoid fever vaccines (Vi, Ty21a) against enteric fever may diminish, which could result in a loss of public confidence and decrease public willingness to be vaccinated.
